# Gender Differences in Nutritional Intake among Rural-Urban Migrants in China

**DOI:** 10.3390/ijerph18189821

**Published:** 2021-09-17

**Authors:** Qian Sun, Xiaoyun Li, Dil Bahadur Rahut

**Affiliations:** 1 College of Economics and Management, Huazhong Agricultural University, Wuhan 430070, China; sunqian0915@webmail.hzau.edu.cn; 2 Asian Development Bank Institute, Tokyo 100-6008, Japan; drahut@adbi.org; 3 Socioeconomics Program, International Maize and Wheat Improvement Center, El Batán, Texcoco 56237, Mexico

**Keywords:** migrants, food consumption, energy intake, nutritional quality, gender, China

## Abstract

Due to rapid economic growth and urbanization, China has witnessed massive migration from rural to urban areas and significant changes in food habits and nutritional intake. This paper empirically examines the factors affecting nutritional intake of 7752 rural-urban migrants and its gender effects, using the China Health and Nutrition Survey (CHNS) data from 1991 to 2011. The descriptive results show that female migrants (FMs) have lower energy intake but have higher proportions of energy from protein and fat than male migrants (MMs), indicating that FMs have a better nutritional quality. The regression results show that the calorie-income elasticities are 0.014 for MMs and 0.018 for FMs. A remarkable positive effect of income on nutritional quality is found for FMs. Employment in non-farm sectors and the community environmental sanitation have a significant increase in calorie intake and nutritional quality, while non-farm employment presents a negative and significant effect on FMs’ calorie intake. Modern market significantly positively affects migrants’ energy intake from protein, thereby improving their nutritional quality. The exogenous switching treatment effect regression results display a significant gender effect on energy intake and its share from protein for migrants, while there is no significant gender effect when it comes to the share of energy from fat.

## 1. Introduction

Ensuring that all people, particularly those most likely to be affected by food insecurity and malnutrition, have equal access to affordable and nutritious food is crucial for human health and welfare. However, sociodemographic characteristics, such as gender, age, education, wealth, and geographic location, show marked differences in nutritional outcomes and malnutrition inequalities [1]. The global prevalence of food insecurity was 25.4% for women and 24% for men, with the largest differences (29.9% vs. 24.8%) found in Latin America [2]. Women also seem more likely to be malnourished and over-nourished. For instance, the prevalence of overweight (BMI ≥ 25) and obesity (BMI ≥ 30) among adults aged 18 and over was 39.2% and 15.1% for women in 2016 compared with 38.5% and 11.1% for men, respectively [1]. Interestingly, women also had a higher prevalence of being underweight (BMI ≤ 18.5). About 9.4% of women are underweight compared to 8.6% of men. Anemia, one form of malnutrition, affected 613.2 million adolescent girls and women of reproductive age, and its prevalence remained high at 32.8% in 2016 [1]. Similar gender differences exist among children and across countries, although this trend is not the same for all countries.

China also shows gender differences in nutritional outcomes of residents. The prevalence of overweight (24 ≤ BMI < 28) and obesity (BMI ≥ 28) among adults was 33.2% and 14.9% for females in 2018 compared with 37.6% and 16.1% for males, respectively [3]. For the adolescent, the prevalence of overweight and obesity for girls was 20.8% and 7.1% in 2016 compared with 35.0% and 15.4% for boys [1]. The proportion of energy provided by protein and fat intake for urban residents was 12.9% and 36.4% in 2015 compared with 11.5% and 33.2% for rural residents, respectively [3]. Rapid urbanization in China is a critical, ongoing trend shaping food-consumption patterns and nutrition transition [4]. A large number of rural residents have moved to urban areas, putting pressure on the urban food system and development. On the other hand, those rural-urban migrants face challenges around accessing nutritious food, adequate employment, sanitation, and health services, all of which affect food consumption and nutrition. There is evidence that the food consumption and nutrients intake of rural-urban migrants are lower than that of local urban residents [5,6], indicating that migrants are a disadvantaged group living in urban areas. There is, therefore, a pressing need to understand drivers of inequality and how they lead to unequal nutritional outcomes.

Given a female’s multiple important roles in food systems, a series of papers have explored the relationship between gender and food security or nutritional outcomes at the household level [7,8,9,10,11,12]. These studies generally agree that female-headed households are more vulnerable to food insecurity than male-headed households and recommend strengthening women’s roles in food production and providing equal access to the resources influencing food security. Contrary to the conventional idea, Aryal et al. [7] and Mallick and Rafi [11] found that there was no significant difference in food security between male- and female-headed households.

At the individual level, substantial previous research has focused on the gender gap in dietary intake and nutritional outcomes of different age groups [13,14,15,16,17,18,19,20,21,22,23,24,25]. General results have demonstrated that females consume less energy and macronutrients and have a relatively worse nutritional status than men. For children, these gender differences have been attributed to physical activities rather than dietary discrimination [14], differential returns to parents from investment in boys and girls rather than parental preference for boys [19]. Furthermore, the nutritional disadvantages for girls are associated with shorter breastfeeding and food allocation inequalities, which leads to greater survival risks compared with boys [18]. For adults, these gender gaps are related to food consumed in terms of quantity and frequency of food consumed [25] and nutritional and healthy food choices [26,27]. In addition, nutritional requirements from a biological perspective [28] as well as time allocation from a sociological perspective [22] clearly reflect the gender gaps in nutrition. Moreover, a recent study reveals that the majority of the gender gaps can be attributed to gender differences in income, education, and social networks [17].

However, the findings on gender and nutrition among adults are mixed and conflicting. Some studies found few gender differences in nutrition, particularly in energy allocation within the household [29], although differential calorie requirements exist due to extremely specialized gender roles in livelihood. Men generally perform works that require physical exertion and hence higher calorie intake compared to females. A recent study indicates that adult women consume more major macronutrients (fat, protein, and carbohydrates) as a percentage of total energy than adult men, although the absolute intake of all macronutrients is lower for women than men [16]. Similar results were presented in Bates et al. [15]. Interestingly, these gender differences in energy and macronutrient intake are moderated by age and, to some extent, by individual socioeconomic status [16].

In summary, gender differences indeed exist in food security and nutrition, whether at the global and regional level or among households and individuals. While gender inequality has caused concern among international organizations, policymakers, and scholars, there is insufficient empirical evidence for underlying drivers of nutritional differences from a gender perspective.

This paper uses longitudinal data from the China Health and Nutrition Survey (CHNS) [30] to examine whether gender differences exist in nutritional intake among adult rural-urban migrants. We then explore the effect of gender on nutritional outcomes. This article makes three novel contributions to the literature. First, unlike other studies, this paper pays close attention to rural-urban migrants, who are disadvantaged groups living in urban areas. This growing special population has been researched less or even neglected in current literature. Therefore, studying this vulnerable group and the urban poor during rapid urbanization is exceptionally important. Second, compared with earlier studies that statistically tested the nutritional gap between two sub-samples separately (e.g., [25]), rather than using a gender dummy indicator variable in the regression (e.g., [11]), we use an exogenous switching treatment regression method, which allows us to examine the heterogeneous effect of rural-urban migrants’ observed or unobserved characteristics on nutritional outcomes. Finally, we select indicators from nutritional quantity (i.e., total energy intake) and quality (i.e., the share of energy from protein and fat), which can better measure individuals’ nutritional intake level. Energy to maintain basic physiological activities can be obtained from the food consumed, and the source of total energy intake is also quite important for understanding nutritional improvement [31]. Previous studies have generally employed absolute intakes of macronutrients or anthropometric indices (like BMI, height-for-age, weight-for-age) to investigate nutrition and health. In contrast, the indicators used in this article, particularly the two nutritional quality indicators, better fit our research focus. Moreover, similar to Bennett et al. [16] and Bates et al. [15], this study may come up with some findings that are different from previous research and generally accepted views.

## 2. Context of the Study Area in China

China has experienced rapid economic growth and urbanization for the last four decades. Thousands of rural residents have migrated to urban areas, substantially increasing the urban population. China’s urbanization rate, calculated as the proportion of the population residing in urban areas against the total population, was 63.89% at the end of 2020, while 45.4% of the total households were registered urban households. This “incomplete urbanization” (a gap of 18.49%) is the result of China’s unique household registration system, called hukou. The rural hukou restricts migrants from gaining full access to public services, medical and health care, and education compared to those registered urban residents. Chen et al. [32] and Han et al. [5] demonstrated that these restrictions, in turn, could reduce total consumption expenditures, including food expenditures for rural-urban migrants, thus affecting food availability and their access to nutrition. Therefore, in the context of China’s household registration system, the vulnerable groups in urban areas (rural-urban migrants) deserve attention.

Furthermore, with accelerated urbanization, large-scale rural-urban migration will continue in the future. Thus, a growing number of migrants will form an important group within the urban population and will have a high probability of changing the urban food system. Compared with the households/individuals staying in rural areas, migrants may have higher incomes and better access to markets and demand more processed and convenience foods as well as animal products, such as meat and eggs. On the other hand, more employment opportunities in urban areas seem to increase the opportunity costs of preparing food and doing housework, thus increasing female labor-force participation [33]. However, as previously mentioned, rural-urban migrants suffer certain inequalities compared to local urban residents due to the constraints of the hukou system. Obviously, the diets and nutrition of rural-urban migrants are different from those of rural-stayed and local urban residents. Given a particularly rapid urbanization setting in China, rural-urban migrants could provide valuable insights into the gender differences in food consumption and nutritional intake.

## 3. Data and Methods

### 3.1. Data

#### 3.1.1. Description of CHNS

The rich data used in this paper were extracted from eight rounds of the China Health and Nutrition Survey (CHNS) for the years 1991, 1993, 1997, 2000, 2004, 2006, 2009, and 2011. A multistage, random cluster process was used to draw the sample from 12 provinces (three provinces, Shaanxi, Yunnan, and Zhejiang, joined since 2015), which differ in topography and socioeconomic status, including income, employment, education, and modernization as well as other related health indicators. The CHNS collected detailed household and individual food consumption information for three consecutive days randomly allocated from Monday to Sunday, allowing us to explore the individual differences in dietary intake and nutritional status.

Household food consumption at home was measured by changes in inventory from the beginning to the end of each survey day. Specifically, well-trained enumerators used Chinese balances to weigh and record all food items in grams, including the initial amount before the survey; the amount of purchased and/or self-produced, discarded, and/or wasted in each survey day; and the total remaining amount at the end of the survey. Thus, the amount of each dish consumed by the household can be obtained for each survey day. Moreover, the CHNS recorded the number of household members and guests at each meal.

Individual foods consumed was recorded for the three consecutive survey days randomly in a week. This was achieved by asking each individual to report all food consumed each survey day through a 24-h recall. Although a few subjects missed one day because of absence, over 99% of the sample supplied dietary intake data for the full three days (see CHNS site). In addition, the amount of individual food consumption can be estimated from the household food consumption and the proportion of each food consumed reported by each member. The combination of the above two methods developed for the CHNS allows us to adjust individual food consumption data if there is a distinct difference. The higher-quality individual food-consumed data enables us to calculate the nutritional intake and analyze the heterogeneity at the individual level. 

To derive our sample, we first classified individuals into four groups based on their household registration type (i.e., hukou: urban or rural) and residence (urban or rural) at the time of the CHNS interviews. Then, we selected the group of people with a rural hukou who were living in urban areas and defined them as rural-urban migrants. We further limited our sample to adults ages 18 years and older because Chinese law sets the legal marriage age at 22 for males and 20 for females (corresponding to 20 and 18 for ethnic minorities, respectively). After screening out the anomalous data, we obtained 7752 observations with complete information, which includes 3609 (46.56%) male rural-urban migrants and 4143 (53.44%) female rural-urban migrants.

#### 3.1.2. Measurement of Nutritional Intake

Nutritional intake is the direct outcome of dietary intake. Based on the Chinese Food Composition Table (FCT 1989, FCT 1991, FCT 2002/2004) [34], we further converted each individual’s dietary intake mentioned above to energy (calories) and nutrients intake. Thus, we computed and obtained the average daily energy and nutrients intake per capita. The Chinese FCT provides the nutritional content for each food item with a unique code, including energy, protein, fat, carbohydrate, and other nutrients. Among them, energy intake is widely used to measure nutritional intake, especially in developing countries. Although energy is necessary for the human body to maintain basic physiological activities, greater energy intake does not mean better nutrient intake. If an individual consumes higher-quality and nutritious but lower-calorie foods, the structure of energy intake could be improved even if the quantity of energy intake decreases [31]. Therefore, Tian and Yu (2015) demonstrated that the source of energy intake was quite important to better understand nutrition improvement [31]. In view of this, we utilized energy intake and its major sources of macronutrients (i.e., protein, fat, and carbohydrate) to characterize individuals’ nutritional status.

Specifically, we employed total energy (calories) intake per capita per day, the share of total energy obtained from protein, and the share of total energy obtained from fat to comprehensively evaluate individual nutritional status. The first indicator indicates the quantity of nutrition, while the other two indices capture the quality of nutrition or the structure of energy intake [31]. According to the Chinese FCT, 1 g of protein provides 4 kilocalories, and 1 g of fat provides 9 kilocalories of energy. The conversion formulas are as follows:

(1) $$Sprotn=\frac{Protein*4}{Energy},\;Sfat=\frac{Fat*9}{Energy}$$

Here *Sprotn* and *Sfat* are separately defined as the shares of the total energy intake per capita per day from protein and fat and are proposed along with total energy intake to measure nutritional status in this study. Obviously, the *Sprotn* and *Sfat* are proportion data ranging from zero to one (excluding 0 and 1). The energy, protein, and fat are daily total energy intake per capita (in kcal/p/d), daily protein intake per capita (in g/p/d), and daily fat intake per capita (in g/p/d), which can be obtained from the CHNS. General findings in current literature suggest that calories from animal-based foods increase, while calories from cereal and starchy staple foods decrease [35,36,37]. In other words, nutritional sources will shift away from carbohydrates to protein and fat with economic growth and social development. Therefore, the shares of total energy intake from protein and fat are expected to increase as socioeconomic status of the people rises, implying an improvement in energy structure and dietary nutrition.

### 3.2. Methods

To explore the gender gap in nutritional intake of migrants, we applied an exogenous switching treatment effect regression (ESTER) model in a counterfactual scenario. The ESTER model has been widely used in research on gender differences in food and nutrition security in developing countries [7,9,10], and it is usually estimated in a two-step procedure.

In the first step, a two-way fixed effects model and a fractional response model were employed to analyze the factors influencing nutritional intake based on the unbalanced panel data. As mentioned above, we used three nutritional indicators as our dependent variables. Specifically, we first utilized fixed-effects (FE) and random-effects (RE) estimators to control for the unobserved individual heterogeneity that may affect energy intake, thereby eliminating many possible sources of endogeneity. Then the Hausman test (presented later) indicated that FE model is more appropriate than RE model. We further controlled time-fixed effects given the existence of observed and unobserved individual-invariant but time-varying heterogeneity. Therefore, the individual- and time-invariant effects model, which is also called the two-way fixed effects model, was estimated in this study. As for the other two dependent variables, both the *sprotn* and *sfat* are continuous and take values between zero and one. Therefore, we conducted a fractional response model, which is suitable for situations where the dependent variable is continuous and restricted to the interval [0, 1] [38] to estimate the drivers of nutritional quality. The regression models are specified as follows:

(2) $$Energy_{it}=\beta_{0}+\sum_{k=1}^{K}\beta_{k}X_{kit}+\lambda_{i}+\mu_{it}$$

(3) $$Sprotn_{it}=F(\alpha_{0}+\sum_{k=1}^{K}\alpha_{k}X_{kit}+\lambda_{i}+\mu_{it})$$

(4) $$Sfat_{it}=F(\theta_{0}+\sum_{k=1}^{K}\theta_{k}X_{kit}+\lambda_{i}+\mu_{it})$$

where F(⋅) in Equations (3) and (4) is assumed to be the logistic function satisfying 0<F(z)<1 for all z∈ℝ. This ensures that the predicted values of the dependent variables lie in the interval (0, 1). $Energy_{it}$, $Sprotn_{it}$, and $Sfat_{it}$ denote the average daily total energy intake, the share of energy obtained from protein intake per day, and the share of energy obtained from fat intake per day of individual *i* at year *t*, respectively. $X_{it}$ is a vector of observed characteristics affecting nutritional status. $\beta_{k}$ represents *k* (*k* = 1, 2, …, *K*) parameters to be estimated. $\lambda_{i}$ is the time-invariant unobserved characteristics of individual *i*, and $\mu_{it}$ is the standard error term.

In the second step of estimation, we applied the ESTER in a counterfactual scenario to estimate the nutritional status of male migrants (MMs) and female migrants (FMs), and then, we compared the expected nutritional status under the actual and counterfactual cases. Following Kassie et al. [9], we present in Table 1 and define below:

(5) $$E\;(NS_{m}|G=1)=\beta_{m}X_{m}$$

(6) $$E\;(NS_{f}|G=0)=\beta_{f}X_{f}$$

(7) $$E\;(NS_{f}|G=1)=\beta_{f}X_{m}$$

(8) $$E\;(NS_{m}|G=0)=\beta_{m}X_{f}$$

where *m* and *f* denote MMs and FMs, respectively. *G* is a dummy variable equal to 1 for MMs and 0 for FMs. Based on Equations (2)–(4), the *NS* captures migrants’ nutritional status, which is measured by the total energy intake per capita per day, and the shares of energy intake from protein and fat. The *X* is a vector of characteristics affecting nutritional intake; the *β* represents a vector of parameters to be estimated. Here, Equations (5) and (6) are the actual nutritional status for MMs and FMs, respectively, which can be observed and estimated directly from the data on the individuals. Equations (7) and (8) are their respective counterfactual expected nutritional status. Using these conditional expectations and considering gender as a treatment variable, we can compute the effect of gender on nutritional status.

Specifically, if MMs’ characteristics had the same coefficients as FMs’ characteristics, then the effect of gender on MMs’ nutritional status (*MMsNS*) would be given as the difference between Equations (5) and (7), which can be presented as follows:

(9) $$MMsNS=E\;(NS_{m}|G=1)-E\;(NS_{f}|G=1)=X_{m}(\beta_{m}-\beta_{f})$$

Similarly, if FMs’ characteristics had the same coefficients as MMs’ characteristics, then the effect of gender on FMs’ nutritional status (*FMsNS*) would be defined as the difference between Equations (6) and (8) and is marked as follows:

(10) $$FMsNS=E\;(NS_{m}|G=0)-E\;(NS_{f}|G=0)=X_{f}(\beta_{m}-\beta_{f})$$

The *MMsNS* and *FMsNS* parameters ($\beta_{m}$, $\beta_{f}$), which are estimated using the fixed-effects model and fractional response model, give the expected nutritional status for MMs and FMs, respectively. Equations (9) and (10) are equivalent to the average treatment effect on the treated and the untreated, respectively, in the impact evaluation literature and the coefficient effects in the literature on wage decomposition.

However, nutritional intake may differ between MMs and FMs even if they have the same observed characteristics or the same coefficients of responding to those individual characteristics. MMs may inherently intake higher calorie foods than FMs due to other endogenous factors influencing energy intake, such as food preference, eating habits, lifestyle, etc. This is called the base heterogeneity (*BH*) and can be derived from Equations (5) and (8) and Equations (7) and (6), as shown below:

(11) $$BH_{m}=E\;(NS_{m}|G=1)-E\;(NS_{m}|G=0)$$

(12) $$BH_{f}=E\;(NS_{f}|G=1)-E\;(NS_{f}|G=0)$$

## 4. Results

### 4.1. Descriptive Statistics

Sample descriptive statistics for rural-urban migrants are reported in Table 2, and multiple methods are employed to test gender differences. MMs make up 46.56% of the total sample, and their average age is about 43 years. Female migrants comprise the other 53.44%, and their average age is 45 years. As shown in the first three rows of Table 2, the energy, protein, and fat intake are significantly higher for MMs than FMs. Specifically, the per capita daily energy intake of MMs is 2412.49 kcal compared to 2003.51 kcal for FMs, which is basically within the range of 1600~2400 kcal recommended by the Chinese Dietary Guidelines. The per capita daily protein and fat intake is 60.55 g and 64.19 g, respectively, for FMs compared to 72.42 g and 73.51 g for MMs. With regards to nutritional quality, FMs have higher proportions of energy from protein and fat than MMs. The average daily shares of energy from protein and fat of the two sub-samples are within the range of 10~20% and 20~30% recommended by the Chinese Dietary Guidelines. Thus, FMs have a higher nutritional quality than MMs. On average, MMs’ nutrients intake (i.e., energy, protein, and fat) is higher than FMs at the 0.01 significance level (*t*-test), but the shares of energy from protein and fat of MMs are slightly lower than that of FMs. This highlights the significant gender gap in nutritional status in absolute terms among migrants in China.

Though the household income per capita for MMs (CNY 8877.7) is slightly higher than FMs (CNY 8794.3), the *t*-statistic shows no significant difference between the two groups. Around 85.7% of MMs are married, while 91.7% of FMs are married. The Pearson chi-square test of independence displays a significant relationship between marital status and gender at the 1% level (chi^2^ = 71.54, *p* = 0.000). The descriptive results also show that about 33% of MMs and 20% of FMs are employed in non-farm sectors, while the remaining migrants account for a large proportion of students, dependents, unemployed, and those farm sectors. This is because we do not exclude students and other non-working respondents. However, there is a distinct gender difference in non-farm employment among migrants at the 1% significance level (chi^2^ = 161.66, *p* = 0.000). Male migrants are more likely to be employed in non-farm jobs than female migrants.

About 33.7% of rural-urban migrants have a completed middle level of education, 21% completed primary education level, and 31% were illiterate. Further gendered disaggregated analysis shows that approximately 39.6% of MMs and 28.5% of FMs have completed middle school. Irrespective of gender, only 1.3% of migrants have a college or higher degree. However, the *p*-value (0.000) of a Mann–Whitney test exhibits a statistically significant difference in education level by gender among migrants. The average number of family members among migrant households is about five members, and the *t*-test for difference in household size between MMs and FMs is insignificant.

As shown in Table 2, the average score of each community component is less than 6 on a scale of 10, indicating that the urbanicity of places where migrants are relocated is of a relatively low level. Specifically, both modern and traditional markets as well as transportation show no significant gap among migrants. However, sanitation conditions are significantly different between gender groups. The *t*-test results show that FMs have moved into better environments and more urbanized communities than MMs.

### 4.2. Nutritional Intake Trends among Migrants

The overall energy intake (in kcal/p/d) of migrants gradually declined by 35% over roughly two decades, from 2638.8 in 1991 to 1726.5 in 2011 (see Table 3). The rate of decline followed a similar pattern for both MMs and FMs. Between 1991 and 2011, the energy consumption was higher for MMs compared to FMs, though the gap narrowed down from 471.8 in 1991 to 273.5 in 2011. During the same period, the share of protein in the total energy intake for the migrants increased gradually from 11.3% in 1991 to 13.6% in 2011, and a similar trend was observed between male and female migrants. The gap in the share of protein was less than 1%, and whether males or females received more energy from protein varies across the year. The share of fat in total energy intake increased from 19.27% in 1991 to 34.41% in 2011. Similar to protein, the gap in the share of fat was less than 1.7% and varies between males and females across the year.

### 4.3. Empirical Results

#### 4.3.1. Factors Influencing Nutritional Intake of Rural-Urban Migrants

To explore the relationship between gender and nutritional intake among adult migrants, we partitioned the sample into FMs and MMs and reported the estimation results on key variables in Table 4. As we illustrated before, we employed individual- and time-fixed effects models to identify the determinants of energy intake per capita per day of migrants and the fractional response model to identify the factors influencing the shares of energy from protein and fat (hereafter abbreviated as *sprotn* and *sfat*, respectively). The models perform well, according to R-squared (0.226, 0.207) and Wald chi-square at the bottom of Table 4.

It is worth mentioning that the fixed-effects estimator is more appropriate to use in the paper, although the Hausman test for MMs group (*p*-value is 0.096) fails to reject the null hypothesis at the 5% significance level. First of all, the fixed-effects estimator is consistent no matter whether the individual effects are related to the explanatory variables. More importantly, the fixed-effects model controls for the estimated bias caused by the correlation between unobserved individual characteristics (food preference, lifestyle, eating habits, etc.) and explanatory variables. Obviously, the assumption of the random-effects estimator that the unobserved heterogeneity is irrelevant to any explanatory variable may be violated in this paper.

As shown in columns (1)–(2), household income has a significant and positive effect on total energy intake for both male and female migrants. The calorie-income elasticities are 0.014 for MMs and 0.018 for FMs. The positive association between income and energy intake indicates that the total energy intake rises as income increases. Additionally, elderly FMs are more likely to consume more calories, while the age coefficient is insignificant for MMs. Married MMs have a higher energy intake than unmarried MMs, showing that being part of a family improves nutritional intake. Employment in non-farm sectors is also associated with a significant increase in calorie intake for MMs at the 5% significance level, while the impact is negative and significant for FMs. Traditional market and community sanitation have a positive and statistically significant effect on total energy intake for all migrants, but there are slight differences in the impact on MMs and FMs. The coefficient for the modern market is positive and significant only for FMs. Additionally, we find that FMs’ energy intake has a distinct decreasing trend with time. The effect of time is also negative but statistically insignificant for MMs.

The fractional response regression results in columns (3)–(6) show that there is a remarkable positive effect of income on total energy provided by protein and fat, respectively, at the 0.01 significance level, except for *sprotn* of MMs. Non-farm employment presents an apparent positive relationship with *sprotn* for all migrants at the 0.01 significance level. Additionally, its impact on *sfat* for MMs and FMs is positive and significant at the 5% and 1% significance level, respectively. Generally, migrants with higher income and those employed in non-farm sectors have a higher share of protein and fat (*sprotn* and *sfat*) intake, indicating a better nutritional quality. The results also show that with an increase in education level, the *sprotn* increases significantly for MMs, and this effect of education is positive and significant for FMs with middle and high school education. In columns (5) and (6), compared with illiterate migrants, those migrants with primary and middle school have significantly higher *sfat*, while those migrants with high school, university, and above education show no significant increase of *sfat*. Household size demonstrates a significant positive effect on the *sprotn* for FMs at 5% significant level, but it has no significant effect for MMs. We also find that there is no significant effect of household size on the *sfat* for MMs and FMs. Modern markets, which means access to supermarkets, restaurants (including fast-food restaurants), and fruit and vegetable stands, show a significant and positive impact on *sprotn* for both MMs and FMs at 10% and 5% significance level, respectively. Traditional markets, which is the distance from the community to various markets, present a positive and significant impact on *sfat* for both MMs and FMs at 5% and 1% significance level, respectively. Transportation infrastructure has a negative and significant effect on *sprotn* of all migrants. Furthermore, community sanitation conditions dramatically affect *sprotn* and *sfat* for all migrants, i.e., positive and significant at the 1% level of significance. We also find that *sprotn* and *sfat* show a clearly increasing trend over time for all migrants, especially for female migrants.

#### 4.3.2. Gender Differences in Nutritional Intake among Rural-Urban Migrants

The ESTER results are presented in Table 5, Table 6 and Table 7 and reveal remarkable gender differences in the nutritional intake of migrants. Cells (a) and (b) show the actual nutritional intake, and cells (c) and (d) show the counterfactual case. In Table 5, we compare the actual values of the expected logarithm of energy intake per day for migrants and observe that this is 0.19 higher for MMs than for FMs. We then converted to the original energy intake per capita per day and found that the gender gap is 398.55 kcal. Similarly, the other two nutritional status indicators, *sprotn* and *sfat*, for MMs are 0.10% and 1.39%, which are lower than those for FMs (compare cells (a) with (b) in Table 6 and Table 7).

However, we cannot obtain the treatment effects of gender on nutritional intake through the simple comparison above [7,9]. Therefore, we compare the actual and counterfactual expected nutritional intake of both groups based on observable and unobservable factors that influence nutritional intake (that is, compare cells (a) with (d) of Table 5, Table 6 and Table 7). Our results in Table 5 show that this gendered energy intake gap would have been increased slightly to 0.015, or 34.29 kcal, if FMs’ observed characteristics had the same coefficients as MMs. Similarly, the gender gap in *sprotn* would have increased to 0.12%. We also conduct a similar comparison under the counterfactual condition (c), as if the MMs’ observed characteristics had the same coefficients as FMs. We find that the gender difference in energy intake per day would have declined to 0.057. By converting to the original value, we find a gender gap of 105.52 kcal. Meanwhile, the gender differences in *sprotn* would have been increased moderately to 0.08%. Overall, under both counterfactual conditions, the results demonstrate that MMs have relatively better energy intake than FMs, and the gender gap would be narrowed to a certain extent for rural-urban migrants.

The last columns in Table 5, Table 6 and Table 7 present the treatment effects of gender on the nutritional intake compared cells (a) with (c) for MMs and cells (d) with (b) for FMs. In the counterfactual case (d), the logarithm of energy intake per day of FMs would have been increased by 0.175. That means the original value is converted to 364.24 kcal if they had had the same coefficients on their observed characteristics as MMs. Similarly, the *sprotn* and *sfat* of FMs would have been declined by 0.22% and 1.50%, respectively, if they had had MMs’ coefficients of observed characteristics. In the counterfactual case (c), MMs’ nutritional status would have been better than it was at the time of study if they had had the same coefficients on their observed characteristics as FMs, with a relatively lower energy intake and higher *sprotn* and *sfat*.

## 5. Discussion

Gender equality in food and nutrition security is not only the inherent right of women and girls but also a key step toward achieving the Sustainable Development Goals (SDGs), particularly ending hunger and improving nutrition (SDG2) for all humanity [39]. Despite SDG commitments to gender equality, women generally have less access to development resources and opportunities than men. If women had the same productive inputs as men, the number of undernourished people could be reduced by 100–150 million [40]. Meanwhile, a large body of literature has demonstrated that empowering women can significantly improve household food security, dietary quality, and children’s nutrition [39,41,42,43,44,45,46]. Generally, in many societies, women play multiple roles in food production, food preparation and allocation, and childcare. These roles may exacerbate gender inequality in nutrition and expose women and girls to a higher risk of food insecurity and poor nutrition [47,48]. In light of this, it is critical that women have access to adequate nutritional foods, which is important for improving household nutrition and a country’s overall human development capacity [49,50,51].

The findings do not consistently support the general assumption from previous literature about the prevalence of gender inequality in terms of diet and nutrition quality in developing countries. With regard to nutritional quantity, we find that female migrants have a lower energy intake than their male counterparts, which supports previous research finding that the nutritional status of females is worse than males [10,13,22]. However, it is worth noting that the nutritional status is better for female migrants than male migrants in terms of nutritional quality, meaning a higher share of energy intake from protein and fat. This finding does not support the general hypothesis that females are a vulnerable group compared to males and would be subjected to detrimental inequalities regarding access to food and nutrition. Similarly, evidence from the UK indicates that women receive a greater proportion of energy from protein and fat than men [16]. From this point of view, females are not entirely disadvantaged in accessing food and nutrition.

A potential explanation for the gender difference in energy intake arises from the differences in migrants’ occupations and labor intensity. This also seems evident in Table 4, which shows that non-agricultural employment significantly increases energy intake for migrants. After moving to urban areas, male migrants without urban hukou often engage in strenuous and heavy physical activities, such as working as a construction worker, miner, or loader, and therefore require more energy to maintain their basic body needs. In contrast, female migrants are usually employed in service industries with relatively lower labor intensity, such as cleaners, cashiers, waitresses, and other jobs. Lower labor intensity is related to less energy consumption so that the proportion of cereal foods consumed is lower in total dietary intake. Using individual dietary intake data, we find that the average percentage of cereals consumed by MMs is 31.09% and higher than that consumed by FMs (28.26%). Therefore, it is not surprising that male migrants consume more calories than their female counterparts.

As previously mentioned, the results of this article on nutritional quality, as measured by the three main macronutrients supplying energy, are contrary to conventional wisdom. This may be attributed to at least three reasons. One possible explanation is that the two groups consume different categories of food. Although female migrants have lower energy intake, they have better energy structure because they consume more nutritious and higher-protein foods, like meat, eggs, and dairy products. These high-value foods can provide more energy per unit than other foods, like grains. In other words, one can reach the same energy level with fewer calories by consuming more protein and fat compared to cereals. Using individual dietary intake data, we also find that the average percentage of the above high-protein foods consumed by FMs is 25.47% and higher than that consumed by MMs (24.75%). Eggs and aquatic products are the main sources of high-quality protein, and they account for 6.60% of average daily total dietary intake for FMs compared with 6.37% for MMs. Dairy and its products account for 9.38% of average daily total dietary intake for FMs compared with 8.24% for MMs. The second interpretation is that women usually are food preparers and caregivers and have better dietary and nutritional knowledge, making them pay closer attention to nutrition in food choices. To a certain extent, education level as a proxy variable of nutrition knowledge presents a significant positive effect on the share of energy from protein for male migrants (Table 4). The ESTER results in Table 6 also indicate that the share of energy from protein for male migrants would have increased significantly under the counterfactual (c). This empirical evidence demonstrates that nutritional knowledge can reasonably explain the findings in nutritional quality. The third possible reason is that female migrants’ social status and time allocation change in the context of urbanization. After migrating to urban areas, the increased opportunity costs of cooking may result in a shift from home-prepared foods toward purchased foods or eating outside the home, especially for female migrants. In urban China, female migrants will spend more time on paid work than unpaid work, like cooking and cleaning, therefore earning income to buy more nutritious food. Although consuming food outside the home may be correlated to high-fat food, females’ desire to maintain body shape and weight drives them to consume healthier and lower-calorie food.

The limitations of this study should be taken into account when analyzing the results. Though the eight-year panel data provides insights into the dynamic changes in the nutritional intake of migrants in China, the nutrition data we used are not the most recent ones due to CHNS database restrictions. However, the findings based on this data are still useful for examining the role of gender in migrants’ nutrition in the context of rapid urbanization in China. Second, our study excludes children ages 0–17, who are also regarded as a vulnerable group. Consequently, the gender gap in nutritional intake may be underestimated. Most previous studies have concluded that children’s food consumption and nutritional outcomes depend on their parents’ behavior or the household head. In light of this, it is appropriate to study children’s nutritional status separately, especially for those left behind in rural areas, which we plan to address in separate subsequent studies. Finally, there is inevitably inaccurate data on food consumed outside the home based on a 24-h dietary recall, which converts to a biased nutritional intake.

Further analysis using this panel data may be needed to examine whether the nutritional intake gap between MMs and FMs persists in different specific age groups and different community urbanization development levels. Using the latter as an example, the market environment affecting food accessibility differs between urban centers and suburban areas. Therefore, it is meaningful to explore whether the gender gaps in nutritional intake are heterogeneous for migrants living in urban centers compared to those residing in the suburbs. This could provide practical suggestions for constructing and improving the food market. Another analysis of food-consumption categories and quantities needs to be conducted in the future. According to the prediction of the United Nations, China’s urbanization rate will reach 70%, and the urban population will increase by 200 million in 2030. Therefore, a better understanding of urbanization effects (including the age and gender structure of the urban population) on dietary patterns will contribute to improved projections of future food demand and supply in urban China. Our findings call for more in-depth research on whether and how migrants’ nutrition changes under the setting of urbanization and the household registration system. In addition, it is worth noting that the proportion of energy from fat (see Table 3, 32.67%) in 2009 exceeded the threshold (30%) set by the 2016 Chinese Dietary Guidelines and the China Food and Nutrition Development Program (2014–2020) and has an increasing trend over the years. This shows us that the overnutrition issues of overweight and obesity should also be concentrated on along with malnutrition and invisible hunger, or micronutrient deficiency.

## 6. Conclusions and Policy Implications

Understanding the role of gender in food security and nutrition and its underlying determinants in developing countries continues to be of interest to international institutions and policymakers, leading efforts to achieve gender equality. Due to China’s rapid urbanization and economic growth, residents’ food consumption and nutrition intake have greatly improved, and the gender gap declined as women’s socioeconomic status and traditional social roles change. However, less attention has been paid to the vulnerable people living in urban areas but holding rural hukou, or rural-urban migrants. This paper investigated the gender gap in nutritional intake for rural-urban migrants and its potential drivers by using unbalanced panel data from the CHNS. We employed the exogenous switching treatment effect regression approach to examine the gender nutritional gap and introduced individual, household, and community characteristics into estimation models.

The empirical results indicate that there is indeed a significant gender gap in nutritional status measured by energy intake and the share of energy from protein among rural-urban migrants. However, no significant gender gap is found when we use the share of energy from fat to measure nutritional status. More importantly, under the counterfactual condition where FMs had the same returns to their observed characteristics as MMs’ characteristics, both energy intake and the share of energy from protein for FMs would have increased and still shown a significant gender gap. This suggests that some inherent gender-related factors, such as physical, function, and physiological structure, make MMs’ energy intake higher than their FM counterparts.

In general, our results also reveal that both nutritional quantity and quality for migrants increase with per capita household income, non-farm employment, and community sanitation conditions. With regard to nutritional quality, a higher level of education improves the share of energy from protein of migrants, particularly male migrants. These results imply that some gender differences may be narrowed or addressed through policy interventions.

This paper suggests that policies should be proposed and implemented to improve health in China. One policy should target rural-urban migrants and other vulnerable groups such as children, the elderly, and the disabled. Another effective policy intervention would promote female participation in urban labor markets and minimize discrimination against females. Besides, this research presents a clear message for policymakers to popularize dietary nutrition knowledge and publish dietary guidelines suitable for different population characteristics. Another policy implication suggests improving access to food markets and building a more urbanized community environment. Finally, there is a need to shift food policy focus away from energy-dense foods toward nutrient-rich foods.

## Figures and Tables

**Table 1:** Conditional expectations, treatment effects, and heterogeneity effects.

Migrant Type	Male Migrants (MMs)	Female Migrants (FMs)	Treatment Effects
Male Migrants	(a) $E\;(NS_{m}|G=1)$	(c) $E\;(NS_{f}|G=1)$	MMsNS=(a − c)
Female Migrants	(d) $E\;(NS_{m}|G=0)$	(b) $E\;(NS_{f}|G=0)$	FMsNS=(d − b)
Heterogeneity Effects	$BH_{m}=(a\;-\;d)$	$BH_{f}=(c\;-\;b)$	

Notes: (1) Cells (a) and (b) denote the nutritional status that is actually observed in a sample; cells (c) and (d) denote the counterfactual nutritional status; (2) G = 1 if the migrant is male; G = 0 if the migrant is female; (3) $NS_{m}$ represents nutritional status indicator for MMs; $NS_{f}$ represents nutritional status indicator for FMs.

**Table 2:** Descriptive Statistics.

Variables	Description of Variables	Total	By Gender of Migrants		Difference (*t*-Stat.) ^a^
			Mean (Male)	Mean (Female)	
Energy	Energy intake per capita per day (kcal/p/d)	2193.914 (748.202)	2412.491 (779.408)	2003.511 (663.722)	408.980 *** (24.678)
Protn	Protein intake per capita per day (g/p/d)	66.077 (26.729)	72.419 (28.179)	60.552 (24.077)	11.867 *** (19.781)
Fat	Fat intake per capita per day (g/p/d)	68.528 (44.331)	73.505 (47.660)	64.193 (40.725)	9.312 *** (9.177)
Sprotn	The share of energy from protein	0.122 (0.028)	0.122 (0.028)	0.123 (0.028)	−0.001 * (−1.653)
Sfat	The share of energy from fat	0.281 (0.125)	0.274 (0.123)	0.287 (0.127)	−0.013 *** (−4.439)
Pcinc	Per capita household income inflated to 2015 (CNY)	8833.117 (11,950.54)	8877.683 (11,645.62)	8794.297 (12,211.29)	83.386 (0.300)
Age	Age of individual (years)	44.068 (15.689)	43.260 (15.294)	44.772 (15.992)	−1.512 *** (−4.237)
Married	Married: 1 = Yes, 0 = No	0.889 (0.314)	0.857 (0.350)	0.917 (0.275)	^b^ 71.540 *** (0.000)
Nonfarm	Nonfarm employment: 1 = Yes, 0 = No	0.26 (0.438)	0.328 (0.469)	0.201 (0.400)	^b^ 161.659 *** (0.000)
Education Level					^c^ −16.776 *** (0.000)
Illiterate	Illiterate: 1 = Yes, 0 = No	0.307 (0.461)	0.212 (0.408)	0.390 (0.488)	
Primary	Primary school: 1 = Yes, 0 = No	0.21 (0.407)	0.214 (0.410)	0.205 (0.404)	
Middle	Middle school: 1 = Yes, 0 = No	0.337 (0.473)	0.396 (0.489)	0.285 (0.451)	
High	High or vocational school: 1 = Yes, 0 = No	0.133 (0.34)	0.164 (0.370)	0.106 (0.308)	
University	University or higher: 1 = Yes, 0 = No	0.013 (0.114)	0.013 (0.115)	0.013 (0.114)	
Hhsize	Household size (number)	4.178 (1.455)	4.197 (1.441)	4.160 (1.466)	0.037 (1.092)
Community Characteristics					
Market	Traditional market score (0–10)	5.662 (3.296)	5.630 (3.287)	5.690 (3.304)	−0.060 (−0.798)
Trans	Transportation score (0–10)	5.531 (2.387)	5.495 (2.398)	5.562 (2.377)	−0.067 (−1.238)
Mart	Modern market score (0–10)	4.414 (2.957)	4.389 (2.968)	4.437 (2.948)	−0.048 (−0.710)
Sani	Community sanitation score (0–10)	5.556 (2.977)	5.428 (2.983)	5.668 (2.966)	−0.240 *** (−3.550)
Year	Dummy variable	-	-	-	
Obs.	Sample size	7752	3609	4143	

Notes: ^a^ The reported *t*-statistics in parentheses are the result of a *t*-test comparing female migrants with male migrants, if not specified; ^b^ the reported chi-square statistics are the result of a Pearson chi-square test for nominal variables comparing female migrants and male migrants. The *p*-values are in parentheses; ^c^ the Mann–Whitney test of education level over gender groups is reported, and the *p*-value is in the parentheses; ***, * significant level at 1% and 10%; standard deviations are reported in parentheses in columns 3–5.

**Table 3:** Changes of average nutritional intake for migrants over 1991–2011.

Year	Energy (kcal/p/d)				Sprotn (%)				Sfat (%)				Sample Size		
	Overall	MMs	FMs	Gap	Overall	MMs	FMs	Gap	Overall	MMs	FMs	Gap	Overall	MMs	FMs
1991	2638.8	2890.0	2418.2	471.8	11.3	11.3	11.2	0.15	19.27	19.0	19.5	−0.48	586	274	312
1993	2487.3	2735.9	2268.6	467.3	11.7	11.6	11.8	−0.17	22.25	21.8	22.6	−0.83	921	431	490
1997	2375.7	2606.0	2166.6	439.4	11.6	11.5	11.7	−0.15	25.65	25.0	26.3	−1.33	1099	523	576
2000	2236.5	2453.3	2046.2	407.1	12.0	11.9	12.1	−0.19	29.76	29.1	30.3	−1.16	1059	495	564
2004	2176.5	2402.7	1983.7	419.0	12.1	12.0	12.2	−0.18	26.56	25.6	27.3	−1.71	941	433	508
2006	2136.7	2344.9	1950.3	394.6	12.0	11.9	12.1	−0.20	29.04	28.4	29.6	−1.13	978	462	516
2009	2030.1	2243.5	1843.8	399.7	12.8	12.8	12.8	−0.04	32.67	31.9	33.3	−1.40	1021	476	545
2011	1726.5	1877.2	1603.7	273.5	13.6	13.7	13.6	0.14	34.41	33.8	34.9	−1.17	1047	515	632

**Table 4:** Estimation results among migrants by male and female.

Variables	Ln(energy)		Sprotn		Sfat	
	(1) MMs	(2) FMs	(3) MMs	(4) FMs	(5) MMs	(6) FMs
Ln(pcinc)	0.014 *	0.018 **	0.007	0.013 ***	0.080 ***	0.061 ***
	(0.007)	(0.008)	(0.005)	(0.005)	(0.012)	(0.012)
Age	−0.013	0.052 **	0.0002	0.0001	0.001	0.002 *
	(0.025)	(0.024)	(0.0004)	(0.0004)	(0.001)	(0.001)
Married	0.062 **	0.037	0.004	0.021	0.009	−0.028
	(0.025)	(0.037)	(0.015)	(0.015)	(0.038)	(0.037)
Nonfarm	0.031 **	−0.030 *	0.049 ***	0.047 ***	0.055 **	0.098 ***
	(0.015)	(0.018)	(0.010)	(0.012)	(0.023)	(0.025)
Education Level (Illiterate)						
Primary	0.006	0.027	0.026 *	0.015	0.049	0.068 **
	(0.028)	(0.027)	(0.013)	(0.013)	(0.039)	(0.032)
Middle	0.027	0.015	0.060 ***	0.024 *	0.095 **	0.057 *
	(0.035)	(0.036)	(0.013)	(0.013)	(0.039)	(0.034)
High	−0.021	0.002	0.060 ***	0.056 ***	0.021	−0.003
	(0.046)	(0.052)	(0.016)	(0.016)	(0.046)	(0.040)
University	0.013	−0.034	0.117 ***	0.053	0.001	−0.085
	(0.083)	(0.122)	(0.044)	(0.040)	(0.080)	(0.083)
Hhsize	−0.008	−0.009	0.005	0.007 **	−0.008	−0.012
	(0.006)	(0.006)	(0.003)	(0.003)	(0.009)	(0.008)
Market	0.005 **	0.005 ***	−0.002	0.001	0.008 **	0.015 ***
	(0.002)	(0.002)	(0.002)	(0.002)	(0.004)	(0.003)
Trans	0.004	−0.0002	−0.004 *	−0.004 **	−0.005	−0.005
	(0.003)	(0.003)	(0.002)	(0.002)	(0.005)	(0.005)
Mart	0.005	0.005 *	0.003 *	0.004 **	−0.007	−0.003
	(0.003)	(0.003)	(0.002)	(0.002)	(0.005)	(0.004)
Sani	0.012 **	0.008 *	0.015 ***	0.015 ***	0.047 ***	0.043 ***
	(0.005)	(0.005)	(0.002)	(0.002)	(0.005)	(0.005)
Year (1991)						
1993	−0.067	−0.174 ***	0.015	0.034 ***	0.083 **	0.099 ***
	(0.055)	(0.055)	(0.015)	(0.012)	(0.039)	(0.037)
1997	−0.105	−0.456 ***	0.002	0.023	0.228 ***	0.284 ***
	(0.152)	(0.149)	(0.016)	(0.014)	(0.041)	(0.039)
2000	−0.161	−0.726 ***	0.019	0.043 ***	0.411 ***	0.456 ***
	(0.225)	(0.224)	(0.016)	(0.014)	(0.043)	(0.041)
2004	−0.140	−0.987 ***	0.045 **	0.075 ***	0.212 ***	0.278 ***
	(0.323)	(0.319)	(0.020)	(0.018)	(0.044)	(0.043)
2006	−0.153	−1.116 ***	0.022	0.048 ***	0.284 ***	0.336 ***
	(0.373)	(0.366)	(0.018)	(0.016)	(0.045)	(0.043)
2009	−0.163	−1.340 ***	0.093 ***	0.102 ***	0.443 ***	0.507 ***
	(0.447)	(0.441)	(0.020)	(0.017)	(0.046)	(0.044)
2011	−0.382	−1.626 ***	0.145 ***	0.144 ***	0.441 ***	0.480 ***
	(0.496)	(0.488)	(0.019)	(0.018)	(0.048)	(0.047)
Constant	8.160 ***	5.850 ***	−2.249 ***	−2.311 ***	−2.290 ***	−2.094 ***
	(0.806)	(0.822)	(0.049)	(0.047)	(0.127)	(0.120)
R^2^ (within)	0.226	0.207				
Log pseudolikelihood			−1252.019	−1437.475	−1973.208	−2301.933
Wald χ^2^ (20)			404.30	511.51	591.79	746.66
*p*-value ^a^	0.096	0.005				
Observations	3394	3876	3394	3876	3394	3876

Note: ^a^ *p*-value is the Hausman test for fixed effects or random effects, indicating the fixed effects is favorable. Robust standard errors in parentheses; *** *p* < 0.01, ** *p* < 0.05, * *p* < 0.1.

**Table 5:** Energy intake, treatment, and heterogeneity effects of migrants.

Migrants Type	Lnenergy		
	MMs	FMs	Treatment Effect
MMs	(a) 7.742	(c) 7.495	0.247 *** (0.015)
FMs	(d) 7.727	(b) 7.552	0.175 *** (0.015)
Heterogeneity effect	0.015 *** (0.005)	−0.057 *** (0.020)	

Note: *** implies significance at less than 1%. Standard errors are in parentheses.

**Table 6:** Share of energy from protein, treatment, and heterogeneity effects of migrants.

Migrants Type	Sprotn		
	MMs	FMs	Treatment Effect
MMs	(a) 0.1212	(c) 0.1230	−0.0017 *** (0.0002)
FMs	(d) 0.1200	(b) 0.1222	−0.0022 *** (0.0002)
Heterogeneity effect	0.0012 *** (0.0002)	0.0008 *** (0.0002)	

Note: *** implies significance at less than 1%. Standard errors are in parentheses.

**Table 7:** Share of energy from fat, treatment, and heterogeneity effects of migrants.

Migrants Type	Sfat		
	MMs	FMs	Treatment Effect
MMs	(a) 0.2759	(c) 0.2913	−0.0154 *** (0.0013)
FMs	(d) 0.2747	(b) 0.2898	−0.0150 *** (0.0013)
Heterogeneity effect	0.0012 (0.0013)	0.0016 (0.0013)	

Note: *** implies significance at less than 1%. Standard errors are in parentheses.

## Data Availability

The data will be made available to the reader upon request.

## References

[1] Global Nutrition Report (2020). Action on Equity to End Malnutrition. North Quay House, Quay Side, Temple Back.

[2] FAO, UNICEF, WFP, WHO (2019). The State of Food Security and Nutrition in the World 2019: Safeguarding Against Economic Slowdowns and Downturns.

[3] Chinese Nutrition Society (2021). Scientific Research Report on Dietary Guidelines for Chinese Residents.

[4] International Food Policy Research Institute (IFPRI) (2017). 2017 Global Food Policy Report.

[5] Han X., Yang S., Chen Y., Wang Y. (2019). Urban segregation and food consumption: The impacts of China’s household registration system. China Agric. Econ. Rev..

[6] Sun Q., Li X., Rahut D.B. (2021). Urbanicity and nutrition: Evidence from rural–urban migrants in China. China Agric. Econ. Rev..

[7] Aryal J.P., Mottaleb K.A., Rahut D.B. (2018). Untangling gender differentiated food security gaps in Bhutan: An application of exogenous switching treatment regression. Rev. Dev. Econ..

[8] Jung N.M., De Bairros F.S., Pattussi M.P., Pauli S., Neutzling M.B. (2016). Gender differences in the prevalence of household food insecurity: A systematic review and meta-analysis. Public Health Nutr..

[9] Kassie M., Ndiritu S.W., Stage J. (2014). What Determines Gender Inequality in Household Food Security in Kenya? Application of Exogenous Switching Treatment Regression. World Dev..

[10] Kassie M., Stage J., Teklewold H., Erenstein O. (2015). Gendered food security in rural Malawi: Why is women’s food security status lower?. Food Secur..

[11] Mallick D., Rafi M. (2010). Are Female-Headed Households More Food Insecure? Evidence from Bangladesh. World Dev..

[12] Teklewold H., Gebrehiwot T., Bezabih M. (2019). Climate smart agricultural practices and gender differentiated nutrition outcome: An empirical evidence from Ethiopia. World Dev..

[13] Abassi M.M., Sassi S., El Ati J., Ben Gharbia H., Delpeuch F., Traissac P. (2019). Gender inequalities in diet quality and their socioeconomic patterning in a nutrition transition context in the Middle East and North Africa: A cross-sectional study in Tunisia. Nutr. J..

[14] Backstrand J.R., Allen L.H., Pelto G.H., Chávez A. (1997). Examining the gender gap in nutrition: An example from rural Mexico. Soc. Sci. Med..

[15] Bates C., Prentice A., Finch S. (1999). Gender differences in food and nutrient intakes and status indices from the National Diet and Nutrition Survey of People Aged 65 Years and Over. Eur. J. Clin. Nutr..

[16] Bennett E., Peters S.A.E., Woodward M. (2018). Sex differences in macronutrient intake and adherence to dietary recommendations: Findings from the UK Biobank. BMJ Open.

[17] Broussard N.H. (2019). What explains gender differences in food insecurity?. Food Policy.

[18] Fledderjohann J., Agrawal S., Vellakkal S., Basu S., Campbell O., Doyle P., Ebrahim S., Stuckler D. (2014). Do Girls Have a Nutritional Disadvantage Compared with Boys? Statistical Models of Breastfeeding and Food Consumption Inequalities among Indian Siblings. PLoS ONE.

[19] Hazarika G. (2000). Gender Differences in Children’s Nutrition and Access to Health Care in Pakistan. J. Dev. Stud..

[20] Kusumaratna R.K. (2016). Gender differences in nutritional intake and status in healthy free-living elderly. Universa Med..

[21] Leblanc V., Bégin C., Corneau L., Dodin S., Lemieux S. (2014). Gender differences in dietary intakes: What is the contribution of motivational variables?. J. Hum. Nutr. Diet..

[22] Padmaja R., Pramanik S., Pingali P., Bantilan C., Kavitha K. (2019). Understanding nutritional outcomes through gendered analysis of time-use patterns in semi-arid India. Glob. Food Secur..

[23] Ren W., Rammohan A., Wu Y. (2014). Is there a gender gap in child nutritional outcomes in rural China?. China Econ. Rev..

[24] Satalić Z., Barić I.C., Keser I. (2007). Diet quality in Croatian university students: Energy, macronutrient and micronutrient intakes according to gender. Int. J. Food Sci. Nutr..

[25] Sudo N., Sekiyama M., Maharjan M., Ohtsuka R. (2005). Gender differences in dietary intake among adults of Hindu communities in lowland Nepal: Assessment of portion sizes and food consumption frequencies. Eur. J. Clin. Nutr..

[26] Arganini C., Saba A., Comitato R., Virgili F., Turrini A., Maddock J. (2012). Gender Differences in Food Choice and Dietary Intake in Modern Western Societies. Public Health—Social and Behavioral Health.

[27] Zugravu C.A., Patrascu D., Prejbeanu I., Rada C. (2009). Gender differences in nutrition and lifestyle attitudes for a sample of Romanians. Ann. Univ. Dunarea De Jos Galati Fascicle VI—Food Technol..

[28] Salmi M. (2018). Nutrition: Gender differences and the role of women. Ital. J. Gend. Specif. Med..

[29] Kimhi A. (2004). Gender Differences in Health and Nutrition in Southern Ethiopia.

[30] CHNS (China Health and Nutrition Survey). https://www.cpc.unc.edu/projects/china/.

[31] Tian X., Yu X. (2015). Using semiparametric models to study nutrition improvement and dietary change with different indices: The case of China. Food Policy.

[32] Chen B., Lu M., Zhong N. (2015). How Urban Segregation Distorts Chinese Migrants’ Consumption?. World Dev..

[33] de Bruin A., Liu N. (2020). The urbanization-household gender inequality nexus: Evidence from time allocation in China. China Econ. Rev..

[34] CDC (Chinese Center for Disease Control and Prevention) (2004). Chinese Food Composition 2004.

[35] Burggraf C., Kuhn L., Zhao Q.-R., Teuber R., Glauben T. (2015). Economic growth and nutrition transition: An empirical analysis comparing demand elasticities for foods in China and Russia. J. Integr. Agric..

[36] Gouel C., Guimbard H. (2018). Nutrition Transition and the Structure of Global Food Demand. Am. J. Agric. Econ..

[37] Zheng Z., Henneberry S.R., Zhao Y., Gao Y. Income Growth, Urbanization, and Food Demand in China. Proceedings of the Agricultural & Applied Economics Association’s Annual Meeting.

[38] Papke L.E., Wooldridge J.M. (2008). Panel data methods for fractional response variables with an application to test pass rates. J. Econom..

[39] International Food Policy Research Institute (IFPRI) (2019). 2019 Global Food Policy Report.

[40] FAO (Food Agriculture Organization of the United Nations) (2011). State of Food and Agriculture 2010-11: Women in Agriculture—Closing the Gender Gap.

[41] Amugsi D.A., Lartey A., Kimani E., Mberu B.U. (2016). Women’s participation in household decision-making and higher dietary diversity: Findings from nationally representative data from Ghana. J. Health Popul. Nutr..

[42] Galiè A., Teufel N., Girard A.W., Baltenweck I., Dominguez-Salas P., Price M., Jones R., Lukuyu B., Korir L., Raskind I. (2019). Women’s empowerment, food security and nutrition of pastoral communities in Tanzania. Glob. Food Secur..

[43] Jones R., Haardörfer R., Ramakrishnan U., Yount K.M., Miedema S., Girard A.W. (2019). Women’s empowerment and child nutrition: The role of intrinsic agency. SSM Popul. Health.

[44] Malapit H.J.L., Kadiyala S., Quisumbing A.R., Cunningham K., Tyagi P. (2015). Women’s Empowerment Mitigates the Negative Effects of Low Production Diversity on Maternal and Child Nutrition in Nepal. J. Dev. Stud..

[45] Sraboni E., Malapit H.J., Quisumbing A.R., Ahmed A.U. (2014). Women’s Empowerment in Agriculture: What Role for Food Security in Bangladesh?. World Dev..

[46] Tsiboe F., Zereyesus Y.A., Popp J.S., Osei E. (2018). The Effect of Women’s Empowerment in Agriculture on Household Nutrition and Food Poverty in Northern Ghana. Soc. Indic. Res..

[47] Brody A., Spieldoch A., Aboud G., Randriamaro Z., Rozel-Farnworth C., Reeves H. (2014). Gender and Food Security: Towards Gender-just Food and Nutrition Security.

[48] Nutrition International (NI) (2018). NI’s Program Gender Equality Strategy. https://www.nutritionintl.org/about/gender-equality-nutrition/.

[49] EU (European Union) (2019). Gender Equality Matters for Nutrition: How EU Development Cooperation can Improve Both Gender Equality and Nutritional Outcomes in the Rural Sector. https://ec.europa.eu/international-partnerships/system/files/gender-equality-matters-for-nutrition-brief-20190412_en.pdf.

[50] Garcia A., Wanner T. (2017). Gender inequality and food security: Lessons from the gender-responsive work of the International Food Policy Research Institute and the Bill and Melinda Gates Foundation. Food Secur..

[51] Oniang’o R., Mukudi E. (2002). Nutrition and gender. Nutrition: A Foundation for Development.

